# Anti-Cancer Effect of Bromelain and Its Combination with Cisplatin on HN5 Cell Line (Squamous Cell Carcinoma)

**DOI:** 10.30476/DENTJODS.2021.89577.1478

**Published:** 2022-09

**Authors:** Marjan Kiani, Ebrahim Zabihi, Shima Nafarzadeh, Hamid Reza Nouri, Ali Bijani, Maryam Seyedmajidi

**Affiliations:** 1 Student Research Committee, Postgraduate Student Dept. of Oral and Maxillofacial Pathology, Babol University of Medical Sciences, Babol, Iran; 2 Cellular and Molecular Biology Research Center, Health Research Institute, Babol University of Medical Sciences, Babol, Iran; 3 Oral Health Research Center, Health Research Institute, Babol University of Medical Sciences, Babol, Iran; 4 Dept. of Immunology, Cellular and Molecular Biology Research Center, Health Research Institute, Babol University of Medical Sciences, Babol, Iran; 5 Social Determinants of Health Research Center, Health Research Institute, Babol University of Medical Sciences, Babol, Iran; 6 Dental Materials Research Center, Health Research Institute, Babol University of Medical Sciences, Babol, Iran

**Keywords:** Apoptosis, Bromelain, Cisplatin, Fibroblast, Oral squamous cell carcinoma

## Abstract

**Statement of the Problem:**

Squamous cell carcinoma (SCC) comprises over 90% of oral malignancies. Cisplatin, as a selective chemotherapy agent to treat SCC, has many side effects despite its high effectiveness. There are some studies on the effects of bromelain derived from pineapple stems on different malignancies.

**Purpose:**

The aim of this study was to investigate the effect of bromelain alone and in combination with Cisplatin on oral squamous cell carcinoma (OSCC) and fibroblast cell lines.

**Materials and Method:**

In this interventional study, the HN5 cell line of OSCC and fibroblast cell line were treated with different concentrations of bromelain alone and in combination with cisplatin. Cell viability test was performed after 24, 48 and 72 hours using MTT (3-)4,5-dimethylthiazol-2-yl(-2,5 diphenyl tetrazolium bromide) assay. In the final stage, the drug-treated cells underwent flow cytometry to assess apoptosis patterns. Data were analyzed using SPSS 17, ANOVA (for general comparison of groups) and LSD post hoc tests (for comparison two groups). *p*< 0.05 was considered statistically significant.

**Results:**

The findings suggested that although bromelain showed toxic effects on HN5 cancer cells, its combination with Cisplatin resulted in little improvement in its effectiveness. Bromelain alone and in combination with Cisplatin presented cytotoxic effects against fibroblasts, which depended on the dosage and time exposure (*p*< 0.05). The flow cytometry results did not support the superior effect of the combination of two medications over Cisplatin alone (*p*> 0.05).

**Conclusion:**

According to the findings, although adding bromelain to Cisplatin reduced toxicity on normal tissues, the combination of these two drugs did not increase the anticancer effect of Cisplatin. Thus, bromelain in combination with Cisplatin is not recommended as an adjuvant drug for OSCC.

## Introduction

Squamous cell carcinoma (SCC) is the most common type of oral cancer with poor prognosis [ 1 ]. The SCC comprising over 90% of oral malignancies is originated from the growth of malignant cells of the stratified squamous epithelium of the oral mucosa [ 2 ]. According to the Center for Disease Control of the Ministry of Health (Iran), SCC is among the 13th most common cancers in Iran [ 3 ]. The risk of being diagnosed with oral and pharynx cancer increases by aging, especially in men [ 2 ]. Though the diagnostic techniques have progressed, SCC has a high incidence in different parts of the world. Recent studies have represented an increased incidence rate of oral squamous cell carcinoma (OSCC) in Iranian young patients [ 3 ]. Oral carcinomas are usually treated with surgery in the primary stages. The intermediate to advanced tumors are usually cured using surgery, radiotherapy, or chemoradiotherapy. When the disease is highly advanced or surgical process fails to deliver acceptable results, patients may be treated by radiotherapy or chemoradiotherapy modalities [ 2 ].

Cisplatin (Cis- [Pt (11) (NH(3))(2)C1(2)] [PtC12 (NH3) or 2 CDDP)) is one of the most effective chemotherapy treatments that is widely used to treat cancer [ 4 ]. Cisplatin (cisdiamminedichloridoplatinum (II) or (CDDP)) is a metallic composition, initially synthesized through M.Peyrone in 1844 and its chemical structure has been firstly discovered by Alfred Werner in 1893 [ 5 ]. Cisplatin as a first-generation anti-cancer combination plays a key role in treatment of different types of cancer; moreover, it is a determining treatment factor in head and neck cancers, especially OSCC, which has been widely used to treat this disease [ 6 - 8 ]. A significant number of patients diagnosed with different types of cancers such as sarcomas, cancers of soft tissue, bones, muscles, and blood vessels have been successfully cured by the use of Cisplatin [ 9 ]. Its mechanism is related to the DNA and transcription inhibition [ 10 ]. However, the usage of Cisplatin is limited due to the side effects on normal tissues [ 11 ].

Pineapple is the common term for *Ananas Comosus*, which is an edible member of *Bromeliaceae* family and has been used as a medical herb in several indigenous cultures [ 12 ]. The crude aqueous extract from stem and fruit of pineapple is known as bromelain. This extract is comprised of different thiol endopeptidases and other components including glucosidases, phosphatases, peroxidases, carbohydrates, celluloses, glycoproteins, and several protease regulators [ 13 ]. Recent studies suggest that bromelain has the ability to modify the key pathways of creating cancer. The anti-cancer activity of bromelain is the direct impact on cancer cells and their microenvironment, as well as the modulation of immune, inflammatory, and hemostatic systems. Probably, the anticancer activity of bromelain is due to its direct effect on cancer cells and their microenvironment, as well as the modulation of immune, inflammatory, and hemostatic systems [ 14 ]. In an experiment by Taussig *et al.* [ 15 ], mouse skin papilloma was treated with bromelain; it was found that bromelain could reduce tumor formation, tumor volume and cause apoptotic cell death. In one study [ 16 ] performed on treatment with bromelain in gastric carcinoma cell lines, KATO III showed a significant reduction in cell growth, while in another study [ 17 ], bromelain reduced the potential for glioblastoma cell invasion and reduced de novo protein synthesis. Bromelain overexpresses the p53 and Bax, which are known as apoptotic activators in the skin of mice [ 18 ]. Bromelain also reduces the activity of cell survival regulators such as Akt and Erk, thus increasing apoptosis in tumors. Various studies have shown the role of NF-κB, Cox-2, and PGE2 as promoters of cancer progression. Evidence suggests that NF-κB signaling and overexpression play an important role in many types of cancer [ 19 - 20 ]. Cox-2, a multifunctional gene from NF-κB, facilitates the conversion of arachidonic acid to PGE2 and thus enhances angiogenesis and tumor progression [ 18 ]. Inhibition of NF-κB, Cox-2, and PGE2 activity is thought to be a potential cancer treatment. Bromelain has been shown to downregulate NF-κB and Cox-2 in rat papilloma and skin tumorigenic models [ 18 ]. Bromelain has also been shown to inhibit NF-κB activity induced by bacterial endotoxin (LPS) as well as the expression of PGE2 and Cox-2 in human monocytic leukemia and murine microglial cell lines [ 21 - 22 ].

Chang *et al.* [ 23 ], showed that bromelain, as part of its antiproliferative mechanisms, increased oxidative stress and superoxide production by 6-fold in bromelain-treated human colon cancer cells. A recent study [ 23 ], also reported the effect of *in vitro* bromelain on macrophage pathway activation and lysosome formation via increasing autophagy-associated proteins levels (ATG5 / 12, beclin, p62, and LC3I / II) leading to apoptosis. The microenvironmental anti-inflammatory properties of bromelain by uncoated cancer cells (depolymerizing MUC-1, fibrin and albumin) through increasing adhesion of lymphocytes to the tumor are thought to expose the tumor to host defense [ 24 ]. Bromelain can neutralize overexpression of the oncoglycoprotein MUC-1, which promotes the proliferation and enhancement of antiapoptotic properties of cancer cells along with invasion and chemical resistance in various human tumors. In addition, bromelain has been shown to cause radiation sensitivity in the 4T1 mouse breast-cancer cell line [ 24 ].

Many vaccine and immunotherapy development programs aim to modulate immune responses. Bromelain is a mixture of cysteine proteases that modulate immune responses [ 25 ]. In a study by Engwerda *et al.* [ 25 ], bromelain was shown to increase the proliferation of accessory cells, T cell receptors (TCRs), and anti-CD28-mediated cell proliferation in the spleen by increasing the stimulatory activity. Despite the increase in T-cell proliferation, bromelain simultaneously reduces IL-2 production in the spleen [ 25 ]. These data suggest that bromelain can inhibit T-cell responses in vivo and *in vitro* simultaneously through a stimulatory effect on accessory cells and a direct inhibitory effect on T-cells. This provides important insights into the immune-modulating activity of bromelain [ 25 ].

In another study by Engwerda *et al.* [ 26 ], bromelain was shown to increase the production of IFN-γ-mediated nitric oxide and TNFα by macrophages. Bromelain can also increase IL-2 and IL-12-mediated IFN-γ production by NK cells [ 26 ]. These results indicate the potential role of bromelain in activating inflammatory responses in situations where individuals may have immunodeficiency [ 26 ].

Fouz *et al.* [ 27 ] investigated the autophagy phenomenon in carcinoma cells of breast under treatment by bromelain and the relationship between autophagy and apoptosis in MCF-7 cells. MCF-7 cells exposed to bromelain represented delayed growth inhibitory response and inductive apoptosis, which was detected using monodansylcadaverine. Apoptotic cell death was clearly discovered [ 26 ]. Pillai *et al.* [ 28 ] evaluated the anti-tumor effects of bromelain in combination with N-acetylcysteine (NAC) and Cisplatin on malignant peritoneal mesothelioma. They have found that a 10mmol/ ml NAC + 75µg/ml bromelain causes cell proliferation inhibition. Gani *et al.* [ 29 ] assessed the anti-proliferation activity of bromelain on colon-cancer cell line in laboratory environment. In their study, different concentrations of bromelain (10, 100 and 1000µg/mlit) were exposed to cancer cells, and cell viability was valuated after 24, 48 and 72 hours using MTT (3-(4,5-dimethylthiazol-2-yl)-2,5 diphenyl tetrazolium bromide) assay. The results have illustrated that bromelain effectively reduces cancer cells proliferation via apoptosis [ 29 ]. Mekkawy *et al.* [ 30 ] presumed that bromelain could act as a radiosensitizer for tumor cells. To prove this hypothesis, MTT cell proliferation *in vitro* was used and showed that pretreatment with bromelain could sensitize irradiated Ehrlich ascites carcinoma (EAC) cells. In their study, animals were divided into five groups and treated with different doses of radiation and bromelain [ 30 ]. Histopathological examination of mice with Ehrlich solid tumor (EST) that underwent gamma radiation and bromelain treatment showed significantly reduced size and weight of the tumors. Therefore, they concluded that bromelain could be considered a radiosensitizer and radioprotector that plays an important role in reducing the radiation dose during radiation therapy [ 30 ]. In addition, Chermahini *et al.* [ 31 ], examined bromelain, Cisplatin, and a combination of bromelain and Cisplatin on PC3 (human prostate cancer cell line), and reported the combination of bromelain and Cisplatin to have a synergistic anti-cancer effect on PC3, which drastically reduces the required dose of Cisplatin. In a study by Murthy *et al.* [ 32 ] on the HepG2 cell line, bromelain was shown to be an anticancer agent with intracellular effects on the expression of P53 and β-catenin proteins. They investigated bromelain in terms of cytotoxicity, cell proliferation, migration, and invasion of the HepG2 cell line in phase S and G2/M cell cycle, and concluded that bromelain inhibits HepG2 cell line tumorigenesis [ 32 ]. Mekkawy *et al.* [ 33 ] examined the combined effect of bromelain and acetylcysteine (BromAc®) in combination with cytotoxic agents such as 5-fluorouracil, gemcitabine, or oxaliplatin on LS174T colon-cancer cell line. Drugs were delivered intraperitoneally, and the animals were monitored for health. Preliminary studies were negative in terms of the effectiveness of oxaliplatin and 5-fluorouracil for tumor growth. Then, Gemcitabine combined with BromAc® was investigated and showed approximately 71% tumor inhibition compared to the control group [ 33 ]. 

The aim of this study was to investigate the effect of bromelain alone and in combination with Cisplatin on HN5 cells of the OSCC. 

## Materials and Method

The HN5 SCC cell line (code CI96) was obtained from Pasture Institute of Iran and transferred to the Amirkola Cellular and Molecular Biology Research Center and their growth medium was promptly changed to RPMI-1640 +FBS10% medium. The HN5 cells had been isolated from SCC of the tongue of a 73 year-old man with tumor stage T2N0M0 and moderate level of differentiation [ 34 ]. The fibroblasts were derived from the prepuce of the newborns with the mean age of 2 months in the operating room of Amirkola Children’s Hospital and Babol Clinic.

Bromelain (SIGMA CODE: B4882) was provided from Sigma-Aldrich company (UK). Before every experiment, 10g powder of bromelain was prepared with sterile phosphate-buffered saline (PBS).

Cisplatin medicine with a concentration of 1mg/ml in 50 ml vial was provided from Mylan trademark form France. Growth medium RPMI-1640 was used to dilute Cisplatin, and in the case of fibroblast, DMEM growth medium was used for medicine dilution. 

At first, 10*10^3^ HN5 cells were seeded into a 96-well plate. Then, control and experimental wells were selected and proper amount of medicine was added to the test well and incubated. After incubation process, MTT powder was mixed in PBS medium, 50ml of the acquired solution was added to the well containing cell lines and medicine, after a 4-hour exposure to 37°C, 150µl of DMSO was added to the mixture and finally was immediately read at 570 nm. At first, different concentrations of bromelain were applied for HN5 cell line, and cell viability was investigated after 24 hours. In the next stage, in addition to the use of different concentrations, mixture concentrations of bromelain and Cisplatin were applied for 8*10^3^ cancerous cells and 1*10^4^ fibroblast during 24, 48 and 72 hours. 

Finally, after a primary review of MTT results, 3 different concentrations were chosen to perform flow cytometry: 75µg/ml of bromelain, 8µg/ml of Cisplatin and 75µg/ml bromelain+8µg/ml Cisplatin. 

At first, cell suspensions were prepared, washed twice by using PBS, centrifuged with 250g rotation speed for 6 min. Next, they were washed with 1ml of 1X buffer (1ml 10X buffer+9ml PBS) and were again centrifuged with 250g rotation speed for 6 min. Later, the cells were suspended in 100µl 1X binding buffer, and 5 µl of Annexin solution was added to the cells. Then, the cells were washed in 1ml of 1X buffer and 200ul of this buffer was poured on the cells. Afterwards, 5µl of PI was added and after 5-10 minutes, flow cytometry analysis was performed. Flow cytometry analysis was performed using the Annexin V Apoptosis Evaluation Kit made in USA, according to factory instructions.

The acquired data were analyzed using SPSS v17, ANOVA tests (for general comparison of groups) and post-hoc LSD tests (for comparison of two groups), and *p*< 0.05 was considered statistically significant [ 35 ].

## Results

**Figure 1:**
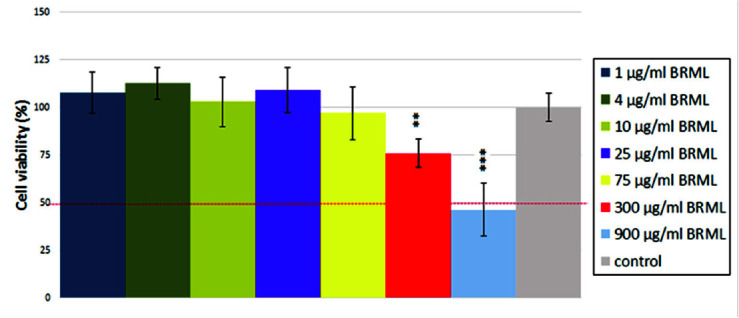
The effects of different concentrations of bromelain on cell viability percentage in the oral squamosu cell carcinoma( OSCC) HN5 cell line after 24 hours. The study was performed using MTT (3-[4,5-dimethylthiazol-2-yl]-2,5 diphenyl tetrazolium bromide) assay method. (BRML: Bromelain) (**: significant difference compared to the control group (*p*< 0.01),significant difference compared to the control group (*p*< 0.001))

**Figure 2:**
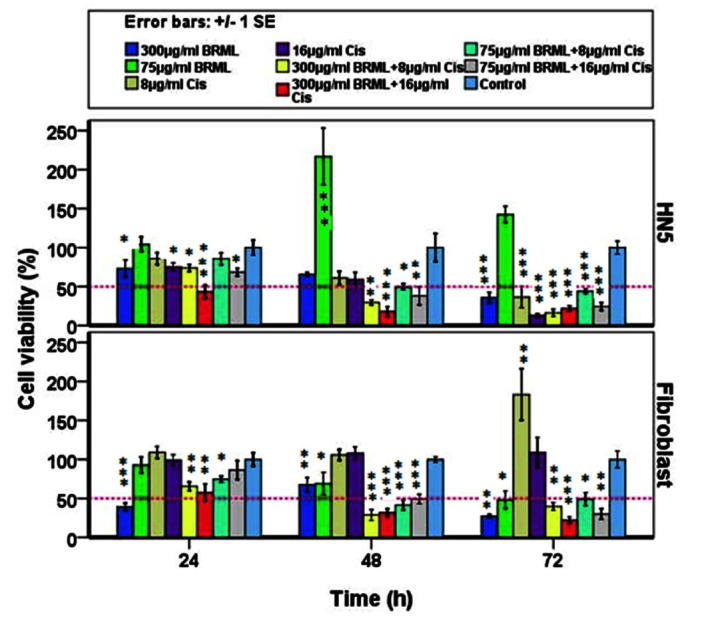
The effects of different concentrations of bromelain and Cisplatin in combination and alone on the cell viability percentage of HN5 and fibroblast cell lines after 24, 48 and 72 hours. The study was conducted using MTT (3-[4,5-dimethylthiazol-2-yl]-2,5 diphenyl tetrazolium bromide) assay method. (BRML: Bromelain, Cis: Cisplatin) (*: Significant difference compared to the control (*p*< 0.05) (**: significant difference compared to the control group (*p*< 0.01), significant difference compared to the control group (*p*< 0.001)

The 1, 4, 10, 25 and 75 µg/ml concentrations of bromelain had no significant effect on cancerous cells (*p*> 0.05) (Figure 1). In the next step, different concentrations of bromelain, Cisplatin and a combination of both were chosen for HN5 cell lines during 24, 48 and 72 hours. The cell viability examination after 24 hours demonstrated that adding 300µg/ml bromelain to 16µg/ml Cisplatin had the most cytotoxicity in cancerous cells and could reduce the cell viability percentage to less than half (*p*= 0.002). Moreover, there was a significant difference between control group and other groups including 300µg/ml bromelain+16µg/ml Cisplatin, 300 µg/ml bromelain+ 8µg/ml Cisplatin and 75µg/ml bromelain+16µg/ml Cisplatin (*p*< 0.001). The cytotoxicity was also found in fibroblasts (*p*< 0.001). Furthermore, the combination concentrations containing 300µg/ml bromelain+8 µg/ml Cisplatin and 75µg/ml bromelain +8µg/ml Cisplatin had significant difference with control group (*p*<0.001). On the other hand, the cytotoxicity of combined concentrations of Cisplatin and bromelain was much higher in fibroblasts. In the last stage, the cells’ life functions in HN5 cell lines and fibroblasts were assessed after 72hours. In this stage, different combined concentrations of medicines reduced the number of cancerous cells to less than half; however, they caused a significant mortality in fibro-blasts, too (*p*< 0.001) (Figure 2). 

**Figure 3:**
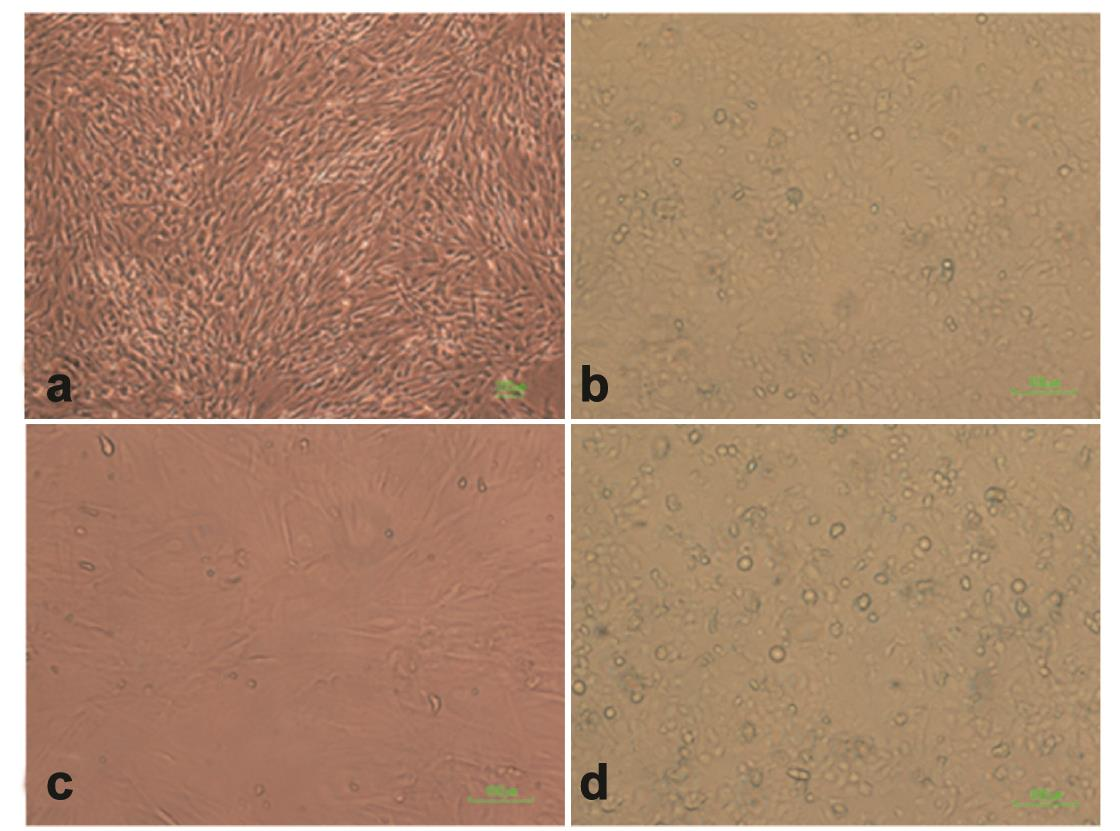
**a:** Microscopic view of fibroblast cells before being treated with medicine, **b:** Microscopic view of HN5 cells before being treated with medicine, **c:** Microscopic view of fibroblast after being treated with 300 μg/ml bromelain, **d:** Microscopic view of HN5 cells after being treated with 300 μg/ml bromelain. Tiny particles represent the cytotoxicity in cells

**Figure 4:**
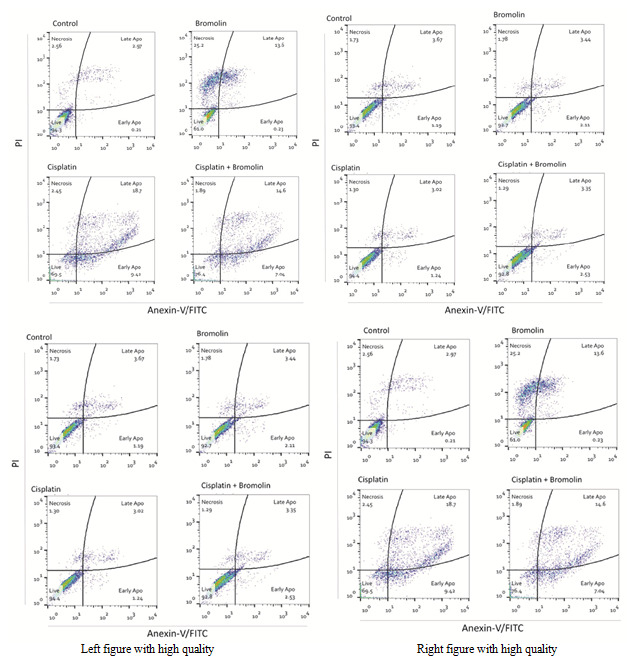
Evaluation of apoptosis amounts using Annexin V/PI in the oral squamous cell carcinoma (OSCC) cell lines HN5 (left) and fibroblast cell lines (right). The cells were exposed to different concentrations of bromelain and Cisplatin in combination and alone after 48 hours; the amounts of apoptosis were measured using flow cytometry and dual-color Annexin V/PI. Live cells are indicated with Annexin V(-)/PI(-), initial apoptosis with Annexin V(+)/PI(-) and dilatory apoptosis or necrosis with Annexin V(+)/PI(+), (75µg/ml bromelain, 8µg/ml Cisplatin, 8µg/ml Cisplatin +75µg/ml Bromelain)

On the microscopic study, a cell deformation was observed so that the cell viability status could be considered (Figure 3). Flow cytometry experiments were conducted to evaluate the effects of different concentrations of medicines on HN5 cell lines and fibroblast without repetition. In flow cytometry, no significant difference between sub-groups of necrosis, apoptosis and live cells were found after 48 hours (Figure 4).

## Discussion

The results showed that bromelain had toxic effects on HN5 cancerous cells so that its combination with Cisplatin did not much improve its effectiveness. Nowadays, the usage of herbal products as a supplementary medicine for curing various illnesses is increasing due to the numerous side effects of chemical medicines [ 18 ]. Meanwhile, considering the prevalence of OSCC and numerous side effects of chemical medicines, the use of herbal medicines or in combination with effective classic medicines may lead to less side effects during the treatment and overcome medical resistance [ 16 , 18 ]. Bromelain is a protease enzymes derived from pineapple essence that has anti-cancer effects such as anticarcinoma in various studies [ 16 , 18 ]. The aim of the present study was to find a proper dosage of bromelain in combination with Cisplatin, which has a toxic effect on cancerous cells and decreases its related side effects on normal cells.

The results showed that bromelain with different concentrations could destroy cancerous cells, as previous studies already represented [ 28 , 31 , 35 ]. Nevertheless, this effect entailed cytotoxicity against normal fibroblasts, which was enhanced in cancer cells and increased with time and increasing doses in both cancer cells and fibroblasts. It was found that bromelain in high concentrations had toxic effects on HN5 cell lines, which is consistent with the results of Amini *et al.* [ 16 ] who found the cytotoxic effects of bromelain on MKN-45, KATO-III cell lines of gastric carcinoma and HT29-5M21, HT29-5F12 cell lines of adenocarcinoma colon. However, they did not investigate the effects of the drugs on normal cells. The present study has suggested that the concentrations of drugs, which can reduce viability of cancer cells to half the initial value, have cytotoxicity effect on fibroblasts even more on cancer cells. Like the current study, Manosroi *et al.* [ 36 ] stated that the cytotoxicity effect of bromelain on cancerous cells was less than that of Cisplatin. 

Besides, bromelain in combination with Cisplatin had no effect on HN5 cell line. Unlike the present results, the findings of Pauzi *et al.* [ 35 ], who investigated the toxicity of the combination of bromelain and Cisplatin, displayed synergistic effects against MDA-MB-231 cells. 

Additionally, Pillai *et al.* [ 28 ] revealed that adding bromelain significantly increased the toxicity of potassium Cisplatin in malignant peritoneal mesothelioma cells. Chermahini *et al.* [ 31 ] examined bromelain, Cisplatin, and a combination of bromelain and Cisplatin on PC3 (human prostate cancer cell line) and reported the combination of bromelain and Cisplatin to have a synergistic anti-cancer effect on PC3, which drastically reduced the required dose of Cisplatin.

In addition, Mohamad *et al.* [ 37 ] examined the anti tumor effect of Cisplatin, bromelain and the combination of Cisplatin and bromelain on 4T1 (triple-negative breast cancer cell line) in mice and evaluated the size of tumors and lung metastases after 28 days. Their results showed that bromelain could potentiate the antitumor effect of Cisplatin on 4T1. The noticeable point here is that none of the studies mentioned [ 27 , 37 ] had employed normal cell groups. All in all, the effects of both medicines especially in high concentrations were more evident after 72 hours. Finally, the concentrations of drugs that had more clear effects were chosen for flow cytometry test. The flow cytometry tests were done 48 hours after treatment of the cells. Overall, there were no significant differences between sub-groups of apoptosis, necrosis, and live cells [ 35 ].

The main difference between the current study and other similar ones [ 27 , 37 ] was the effect of using synchronous medicines on cancerous cell lines and normal fibroblast. Although some of the processes occurring in the cancer progression such as cell proliferation and apoptosis are dependent on proteases, the proteolytic activity in tumors is regulated in a complex manner, because the cancer and stroma cells including fibroblasts, endothelial and inflammatory cells are involved [ 38 ]. However, clinical trials using protease inhibitors have far been unsuccessful except for a few applications of matrix metalloprotease (MMP) inhibitors when used in combination with cytostatic anticancer agents and/or in the early stages of cancer [ 38 ].

Since the proteolytic effects of bromelain have been reported *in vitro* and no strong evidence is available for in vivo effects, and concerning that most *in vitro* studies have been performed without considering normal cells (such as normal fibroblasts), it seems that bromelain-induced cytotoxicity has a direct proteolytic effect and is not a unique mechanism to counter cancer cells. This confirms that bromelain causes more cytotoxicity on normal cells than cancerous ones.

At the end, it can be concluded that even though bromelain can increase the toxicity of Cisplatin against HN5 cell lines of the OSCC in some concentrations outside of the body, numerous side effects are appeared with these medicines due to non-selective toxicity. This finding in addition to the enzyme-protein structure of bromelain makes its probable prescription more difficult. Moreover, it does not appear this medicine is a proper choice for clinical studies to enhance the Cisplatin's effects.

## Conclusion

Bromelain had toxic effects on HN5 cancerous cells and its combination with Cisplatin did not significantly improve its effectiveness. Bromelain alone and in combination with Cisplatin presented cytotoxic effects against fibroblasts, in which these effects were depended on the dosage and time exposure. The acquired results of flow cytometry did not justify the better effects of the combination of two medicines compared to the Cisplatin alone. It seems that the use of bromelain in combination with Cisplatin does not help reduce the dose of Cisplatin in chemotherapy for squamous cell carcinoma.

## Acknowledgement

The authors would like to thank the personnel of the Cellular Molecular Research Center, especially Mrs. Masoumeh Ghasemi who managed the experiments in the laboratory. The Vice Chancellor of Research and Technology in the Babol University of Medical Sciences funded this study.

## Conflict of Interest

 The authors declare that they have no conflict of interest.
